# Our New York Letter

**Published:** 1888-08

**Authors:** 


### OUR NEW YORK LETTER.

THE TREATMENT OF STRICTURE BY ELECTRICITY—NEWMAN'S METHOD ENDORSED—INTERNATIONAL CONGRESS OF MEDICAL JURISPRUDENCE—HIGH STANDARD OF MEDICAL EDUCATION IN THE COLLEGE OF PHYSICIANS AND SURGEONS.

*Editors Atlanta Medical and Surgical Journal :*

The method of treating urethral strictures by electricity has not been very extensively carried on by surgeons here, probably owing to their lack of knowledge on this subject, and also to skepticism regarding the results said to be obtained. Dr. Thos. H. Burchard has become quite an enthusiast on the treatment of these strictures by electrolysis, and his paper, read before the Northwestern Medical and Surgical Society, has aroused considerable interest. He employed Dreicher's forty-cell battery, containing carbon and zinc elements, and also Newman's olivary insulated bougies. His first patient mentioned had two strictures, the first admitting a 12 French instrument and the second a number 7. A Newman's bougie, No. 14, was passed to the first stricture and with a current of six cells, the instrument slipped through the stricture in fifteen minutes. Both strictures were treated in this manner at various intervals of time, and in about two months no stricture could be appreciated by Otis' urethrometer and a 30 sound passed easily. In another case, the patient had been subjected to internal urethrotomy twice. Examination revealed one stricture admitting a No. 9 bougie and another three inches from the meatus, an inch long, barely admitting a No. 6. A numbered insulated bougie was connected with the negative pole and the positive was placed over Scarpa's triangle. The current being gradually increased till ten cells were in use, the instrument slipped through the stricture in eight minutes. The size of the insulated bougies was gradually increased until No. 30 passed readily and the patient was cured so far as the presence of stricture could be ascertained. Seventeen other cases were treated. The applications averaged about 10 minutes for each stricture and from 5 to 14 cells were used. In some of the cases a temporary contraction of the stricture occurred a few days after electrolysis. The lining membrane of the urethra was left in an unusually smooth condition after treatment by this method, as shown by the sensation felt in passing a sound. Dr. Burchard regards strictures, in which the fibrous element predominates, as most amenable to this treatment. The operation, he believes, is radical and effective physiologically and does not aim at cauterization. In his experience it is a painless and bloodless procedure and practically devoid of danger from sepsis.

Dr. Robert Newman took part in the discussion and said that cases properly treated by the electric current did not relapse. The current should never, he said, be used if there is any inflammation of the urethra, and in cases where pain is complained of, it will generally be found that this rule has been violated. A discharge from the urethra during the first few days after treatment sometimes occurred, but there should be no irritability of the bladder.

Dr. Powell had performed external urethrotomy on a patient said to have been cured by electricity under Dr. Newman. Dr. Powell asked if the current destroyed the mucous membrane, or if a perfectly normal tissue remained. Dr. Wood remembered when Dr. Newman illustrated the effect of the current on a piece of cartilage covered with mucous membrane. The cartilage became dissolved into its original constituents, but the mucous membrane remained unchanged. In speaking of urethral stricture, he said urethrotomy destroys the ring at one point only, while electrolysis removed the entire ring. Dr. Dessau mentioned a case which he attempted to cure by electricity. Œdema of the prepuce and swelling of the penis was caused, but one stricture was cured. Dr. Page in one case had seen the current produce so much pain that the patient refused to submit to further applications. The closing remarks were made by Dr. Newman, who said that two hundred cases which he reported had remained cured for from three to eleven years. He had never seen destruction of the mucous membrane caused. In his opinion, the electrode employed by Dr. Dessau must have been poorly insulated, allowing the current to escape not only at its end, but along the stem also. At present Dr. Newman uses the galvanometer and a current varying from three to five milliampéres.

The Medico-Legal Society, of this city, has decided to try and bring about an International congress of medical jurisprudence, to be held in this country in June, 1889. It is intended to have all nations represented, or to contribute papers for discussion.

It is also their intention to nationalize the Medico-Legal Society by enlarging its membership so as to include every State and Territory of the Union. In a circular issued by the Society it is stated that medicine and jurisprudence should unite closer for a clearer understanding of the principles in both these branches of learning in the exercise of functions requiring practical application in the government of society. This, it is claimed, is the special field of medico-legal science and calls for the most intimate relationship between medicine and the law. It is said that the Society has members in every State but eight, and in all the Territories except four, so steps will be taken to secure the aid of prominent men in the localities not represented, who will take an interest in this work. A vice-president is to be elected from each State and members will be asked to report all interesting cases to the Society. Over one hundred persons have lately joined the Society, and among this number are about twenty-five superintendents of insane asylums outside of New York. Several cash prizes for original essays will be given this year by the Society and, from all appearances, a rapidly growing interest is being taken in medico-legal science.

The College of Physicians and Surgeons has decided to require an entrance examination for the session of '88-'89 and thereafter. The requirements will probably appear formidable enough to frighten away a good many students, but the general impression here is that the college is pursuing the proper course. Hereafter applicants will have to undergo a written examination on the following branches before matriculating:

English composition on some announced subject; the translation of simple Latin sentences from certain Roman authors; Arithmetic in all its branches; Algebra through simple equasions; Plane Geometry.

As in other college entrance-examinations, a candidate may pass successfully, or be conditioned, or rejected. The annual course will cover between eight and nine months, but at the same time the number of lectures will not be increased. Hereafter the course of study will be spread over a period of three years, according to the curriculum of the college. This includes, besides other things, practical work in histology, physiological and medical chemistry, pathology and pathological Histology. Otherwise the curriculum is about as usual. In the examinations for the degree of M. D. the clinical professors are each to give one question, a privilege which they have never before had. There will be exceptions made in most all these above mentioned rules, as, for example, where a candidate for admission is a graduate from a reputable academic college, no preliminary examination is required. A. F.

*New York, July 8th, 1888.*

